# Crystal structure of 4-methyl-*N*-[2-(piperidin-1-yl)eth­yl]benzamide monohydrate

**DOI:** 10.1107/S2056989015007653

**Published:** 2015-04-30

**Authors:** B. K. Revathi, D. Reuben Jonathan, S. Sathya, K. Prathebha, G. Usha

**Affiliations:** a PG and Research Department of Physics, Queen Mary’s College, Chennai-4, Tamilnadu, India; b Department of Chemistry, Madras Christian College, Chennai-59, India

**Keywords:** crystal structure, piperidine, benzamide, hydrogen bonding

## Abstract

In the title compound, C_15_H_22_N_2_O·H_2_O, the dihedral angle between the planes of the piperidine and benzene rings is 31.63 (1)°. The piperidine ring adopts a chair conformation. The water solvent mol­ecule is involved in inter­species O—H⋯O, O—H⋯N, N—H⋯O and weak C—H⋯O hydrogen-bonding inter­actions, giving rise to chains extending along [010].

## Related literature   

For the biological activity of piperidine and benzamide derivatives, see: Ramalingan *et al.* (2004 ▸); Sargent & May (1970 ▸); Magar *et al.* (2010 ▸); Fun *et al.* (2011 ▸); Haffner *et al.* (2010 ▸); Lavanya *et al.* (2010 ▸). For related structures, see: Ávila *et al.* (2010 ▸); Prathebha *et al.* (2014 ▸, 2015 ▸); Al-abbasi *et al.* (2010 ▸). For the synthesis, see: Prathebha *et al.* (2014 ▸, 2015 ▸).
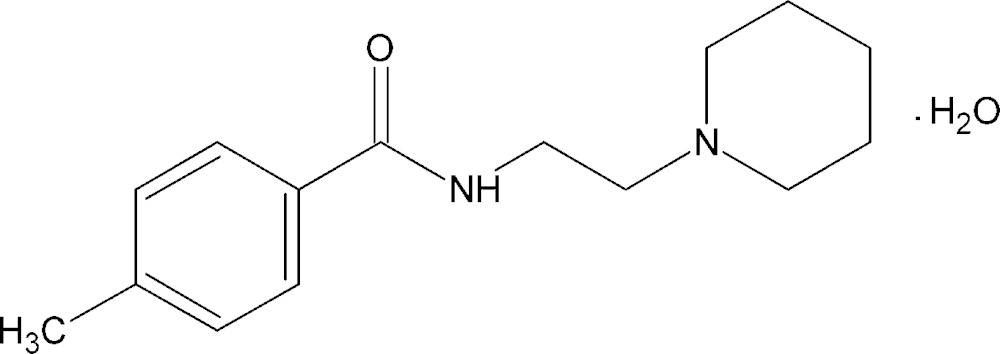


## Experimental   

### Crystal data  

C_15_H_22_N_2_O·H_2_O
*M* *_r_* = 264.36
Monoclinic, 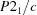

*a* = 14.8504 (17) Å
*b* = 6.8243 (6) Å
*c* = 15.0070 (18) Å
β = 98.653 (4)°
*V* = 1503.6 (3) Å^3^
*Z* = 4
Mo *K*α radiation
μ = 0.08 mm^−1^
*T* = 293 K
0.24 × 0.22 × 0.22 mm

### Data collection  

Bruker Kappa APEXII CCD diffractometer
Absorption correction: multi-scan (*SADABS*; Bruker, 2004 ▸) *T*_min_ = 0.980, *T*_max_ = 0.986
25938 measured reflections
3735 independent reflections
2311 reflections with *I* > 2σ(*I*)
*R*_int_ = 0.031

### Refinement  

*R*[*F* ^2^ > 2σ(*F* ^2^)] = 0.056
*wR*(*F* ^2^) = 0.203
*S* = 1.11
3688 reflections
181 parameters
3 restraints
H atoms treated by a mixture of independent and constrained refinement
Δρ_max_ = 0.34 e Å^−3^
Δρ_min_ = −0.22 e Å^−3^

### 

Data collection: *APEX2* (Bruker, 2004 ▸); cell refinement: *APEX2* and *SAINT* (Bruker, 2004 ▸); data reduction: *SAINT* and *XPREP* (Bruker, 2004 ▸); program(s) used to solve structure: *SHELXS97* (Sheldrick, 2008 ▸); program(s) used to refine structure: *SHELXL97* (Sheldrick, 2008 ▸); molecular graphics: *ORTEP-3 for Windows* (Farrugia, 2012 ▸); software used to prepare material for publication: *SHELXL97*.

## Supplementary Material

Crystal structure: contains datablock(s) I, New_Global_Publ_Block. DOI: 10.1107/S2056989015007653/zs2330sup1.cif

Structure factors: contains datablock(s) I. DOI: 10.1107/S2056989015007653/zs2330Isup2.hkl

Supporting information file. DOI: 10.1107/S2056989015007653/zs2330Isup3.cml

**Fig. 1.**
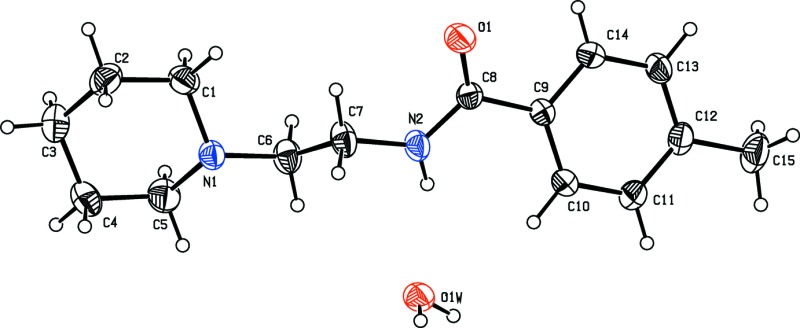
The molecular structure of the title compound, with displacement ellipsoids drawn at the 30% probability level.

. DOI: 10.1107/S2056989015007653/zs2330fig1.tif
The mol­ecular structure of the title compound, with displacement ellipsoids drawn at the 30% probability level.

**Fig. 2.**
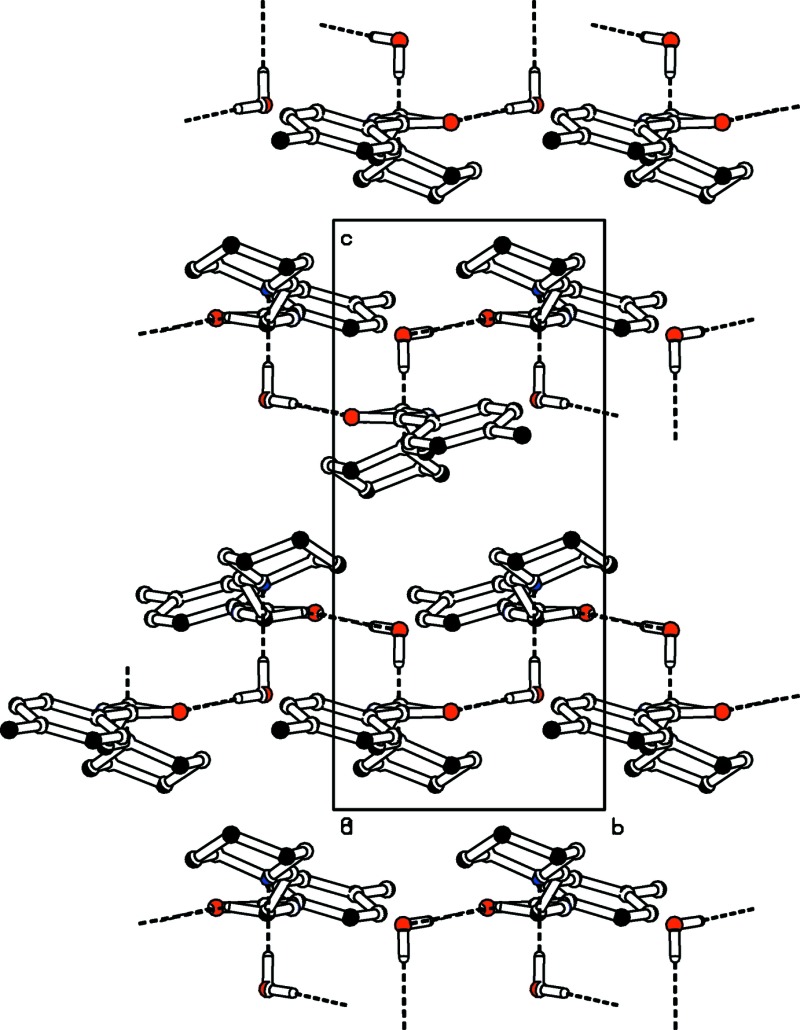
The packing of the molecules in the crystal structure. The dashed lines indicate hydrogen bonds.

. DOI: 10.1107/S2056989015007653/zs2330fig2.tif
The packing of the mol­ecules in the crystal structure. The dashed lines indicate hydrogen bonds.

CCDC reference: 1054604

Additional supporting information:  crystallographic information; 3D view; checkCIF report

## Figures and Tables

**Table 1:** Hydrogen-bond geometry (, )

*D*H*A*	*D*H	H*A*	*D* *A*	*D*H*A*
O1*W*H1*W*O1^i^	0.85(2)	1.99(2)	2.840(2)	177(3)
O1*W*H2*W*N1^ii^	0.85(2)	2.04(2)	2.883(3)	177(3)
N2H2O1*W*	0.86	2.11	2.906(2)	153
C7H7*A*O1*W* ^iii^	0.97	2.55	3.472(3)	159
C10H10O1*W*	0.93	2.51	3.374(3)	154

Symmetry codes: (i) 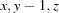; (ii) 
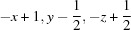
; (iii) 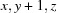.

## supplementary crystallographic information

### S1. Comment

Biologically active alkaloids of substituted piperidines have been targeted for their total or partial synthesis (Ramalingan *et al.*, 2004). Piperidines are known to have *CNS* depressant action at low dosage levels and stimulant activity with increased doses. In addition, the nucleus also possesses analgesic, anglionic blocking and anesthetic properties as well (Sergeant & May, 1970). Benzamides have been reported to correlate with many pharmacological processes such as anti-emetic, anti-psychotic and anti-arrythmic activities. Various *N*-substituted derivatives of benzamide are reported to possess anti-convulsant activity (Magar *et al.*, 2010; Fun *et al.*, 2011). Recently, Haffner & Ulrich (2010) reported that some *N*-substituted derivatives of benzamide can block the Kv1.3 ion channel. Moreover, these have been scanned for anti-microbial and anti-oxidant activities (Lavanya *et al.*, 2010).

The substituted benzamide derivative, the title compound, C_15_H_24_N_2_O_2_, has been prepared and the structure is reported herein. In this compound (Fig. 1) the dihedral angle between piperidine ring the and the benzene ring ring is 31.63 (1)°. The C—C, C—N and C═O bond lengths and C—C—C and C—N—C bond angles are in the normal range and are comparable with literature values and are also in good agreement with the values in similar reported structure (Avila *et al.*, 2010, Prathebha *et al.*, 2014). The C═O distance [1.231 (2) Å] is comparable with a previously reported value (Al-abbasi *et al.*, 2010)·. The bond angle sum around N1 [330.45 (2)°], shows *sp*^3^ hybridization of the atom. The piperidine ring adopts a chair conformation with puckering parameters of q2 = 0.035 (3) Å, φ2 = 182 (5)° q3 = -0.564 (3) Å, QT = 0.565 (3)0143 (2) Å and θ2 = 176.9 (3)°.

The water molecule is involved in the formation of inter-species hydrogen-bonding interactions (Table 1), acting as both a double donor (O1*W*—H···O^i^ and O1*W*—H···N1^ii^) as well as an acceptor (N2—H···O1*W*. One-dimensional chains are generated, extending along [010] (Fig. 2). Weak C—H···O*W* hydrogen bonds are also present.

### S2. Experimental

The title compound was synthesized following a published procedure (Prathebha *et al.*, 2014, 2015). In a 250 ml round-bottomed flask, 120 ml of ethylmethylketone was added to 1,2-aminoethylpiperidine (0.02 mol) and stirred at room temperature. After 5 min, triethylamine (0.04 mol) was added and the mixture was stirred for 15 min. 4-Methylbenzoyl chloride (0.04 mol) was then added and the reaction mixture was stirred at room temperature for 2 hr. A white precipitate of triethylammonium chloride was formed, which was filtered and the filtrate was evaporated to give the crude product. Two recrystallizations from ethylmethylketone give colourless block-like crystals of the title compound (yield: 82%).

### S3. Refinement

Hydrogen atoms were positioned geometrically and treated as riding on their parent atoms and water H-atoms were located from difference Fourier maps and refined with C—H distance of 0.93–0.97 Å, an O—H distance of 0.85 (2) Å and an N—H distance of 0.86 Å, with *U*_iso_(H) = 1.5 *U*_eq_(C-methyl), 1.5*U*_eq_(O) and 1.2*U*_eq_(C) for other H atoms. One reflection (100) was considered to be affected by the beamstop.

### Crystal data

**Table:**

C_15_H_22_N_2_O·H_2_O	*F*(000) = 576
*M**_r_* = 264.36	*D*_x_ = 1.168 Mg m^−^^3^
Monoclinic, *P*2_1_/*c*	Mo *K*α radiation, λ = 0.71073 Å
*a* = 14.8504 (17) Å	θ = 1.4–28.3°
*b* = 6.8243 (6) Å	µ = 0.08 mm^−^^1^
*c* = 15.0070 (18) Å	*T* = 293 K
β = 98.653 (4)°	Block, colourless
*V* = 1503.6 (3) Å^3^	0.24 × 0.22 × 0.22 mm
*Z* = 4	

### Data collection

**Table:**

Bruker Kappa APEXII CCD diffractometer	3735 independent reflections
Radiation source: fine-focus sealed tube	2311 reflections with *I* > 2σ(*I*)
Graphite monochromator	*R*_int_ = 0.031
ω and φ scans	θ_max_ = 28.3°, θ_min_ = 1.4°
Absorption correction: multi-scan (*SADABS*; Bruker, 2004)	*h* = −19→19
*T*_min_ = 0.980, *T*_max_ = 0.986	*k* = −9→9
25938 measured reflections	*l* = −19→20

### Refinement

**Table:**

Refinement on *F*^2^	Primary atom site location: structure-invariant direct methods
Least-squares matrix: full	Secondary atom site location: difference Fourier map
*R*[*F*^2^ > 2σ(*F*^2^)] = 0.056	Hydrogen site location: inferred from neighbouring sites
*wR*(*F*^2^) = 0.203	H atoms treated by a mixture of independent and constrained refinement
*S* = 1.11	*w* = 1/[σ^2^(*F*_o_^2^) + (0.0894*P*)^2^ + 0.5035*P*] where *P* = (*F*_o_^2^ + 2*F*_c_^2^)/3
3688 reflections	(Δ/σ)_max_ < 0.001
181 parameters	Δρ_max_ = 0.34 e Å^−^^3^
3 restraints	Δρ_min_ = −0.22 e Å^−^^3^

### Special details

**Table:**

Geometry. All e.s.d.'s (except the e.s.d. in the dihedral angle between two l.s. planes) are estimated using the full covariance matrix. The cell e.s.d.'s are taken into account individually in the estimation of e.s.d.'s in distances, angles and torsion angles; correlations between e.s.d.'s in cell parameters are only used when they are defined by crystal symmetry. An approximate (isotropic) treatment of cell e.s.d.'s is used for estimating e.s.d.'s involving l.s. planes.
Refinement. Refinement of *F*^2^ against ALL reflections. The weighted *R*-factor *wR* and goodness of fit *S* are based on *F*^2^, conventional *R*-factors *R* are based on *F*, with *F* set to zero for negative *F*^2^. The threshold expression of *F*^2^ > σ(*F*^2^) is used only for calculating *R*-factors(gt) *etc*. and is not relevant to the choice of reflections for refinement. *R*-factors based on *F*^2^ are statistically about twice as large as those based on *F*, and *R*- factors based on ALL data will be even larger.

### Fractional atomic coordinates and isotropic or equivalent isotropic displacement parameters (Å^2^)

**Table:**

	*x*	*y*	*z*	*U*_iso_*/*U*_eq_	
C1	0.38752 (16)	0.4342 (3)	0.07521 (17)	0.0542 (6)	
H1A	0.3939	0.4274	0.0119	0.065*	
H1B	0.4338	0.5227	0.1046	0.065*	
C2	0.29416 (17)	0.5141 (4)	0.08428 (19)	0.0636 (7)	
H2A	0.2896	0.5320	0.1476	0.076*	
H2B	0.2861	0.6408	0.0549	0.076*	
C3	0.22041 (17)	0.3770 (4)	0.04252 (19)	0.0665 (7)	
H3A	0.1619	0.4229	0.0553	0.080*	
H3B	0.2186	0.3751	−0.0224	0.080*	
C4	0.23786 (17)	0.1739 (4)	0.0794 (2)	0.0657 (7)	
H4A	0.2303	0.1724	0.1425	0.079*	
H4B	0.1936	0.0845	0.0473	0.079*	
C5	0.33188 (17)	0.1055 (4)	0.0704 (2)	0.0635 (7)	
H5A	0.3415	−0.0241	0.0966	0.076*	
H5B	0.3377	0.0961	0.0070	0.076*	
C6	0.49185 (15)	0.1599 (4)	0.10743 (17)	0.0541 (6)	
H6A	0.5052	0.1855	0.0472	0.065*	
H6B	0.4909	0.0190	0.1156	0.065*	
C7	0.56594 (14)	0.2458 (4)	0.17469 (18)	0.0558 (6)	
H7A	0.5700	0.3857	0.1646	0.067*	
H7B	0.5519	0.2259	0.2351	0.067*	
C8	0.72909 (13)	0.2522 (3)	0.16379 (13)	0.0409 (5)	
C9	0.81183 (13)	0.1325 (3)	0.15644 (13)	0.0386 (4)	
C10	0.82043 (14)	−0.0617 (3)	0.18282 (15)	0.0471 (5)	
H10	0.7727	−0.1231	0.2054	0.057*	
C11	0.89864 (15)	−0.1651 (3)	0.17615 (16)	0.0517 (6)	
H11	0.9028	−0.2954	0.1945	0.062*	
C12	0.97060 (14)	−0.0808 (4)	0.14315 (14)	0.0500 (5)	
C13	0.96249 (15)	0.1133 (4)	0.11755 (16)	0.0553 (6)	
H13	1.0104	0.1739	0.0949	0.066*	
C14	0.88495 (15)	0.2194 (3)	0.12477 (15)	0.0500 (5)	
H14	0.8818	0.3509	0.1082	0.060*	
C15	1.05545 (18)	−0.1956 (5)	0.1353 (2)	0.0762 (8)	
H15A	1.0492	−0.3265	0.1571	0.114*	
H15B	1.1069	−0.1334	0.1706	0.114*	
H15C	1.0645	−0.2002	0.0734	0.114*	
N1	0.40171 (11)	0.2394 (2)	0.11529 (12)	0.0452 (4)	
N2	0.65238 (11)	0.1538 (3)	0.16645 (13)	0.0497 (5)	
H2	0.6540	0.0281	0.1631	0.060*	
O1	0.73327 (11)	0.4323 (2)	0.16610 (13)	0.0613 (5)	
O1W	0.61401 (11)	−0.2569 (2)	0.19557 (13)	0.0553 (5)	
H1W	0.6502 (18)	−0.350 (4)	0.189 (2)	0.090 (10)*	
H2W	0.611 (2)	−0.259 (5)	0.2514 (13)	0.116 (14)*	

### Atomic displacement parameters (Å^2^)

**Table:**

	*U*^11^	*U*^22^	*U*^33^	*U*^12^	*U*^13^	*U*^23^
C1	0.0519 (13)	0.0525 (13)	0.0580 (13)	−0.0099 (10)	0.0078 (10)	0.0021 (10)
C2	0.0657 (16)	0.0500 (13)	0.0732 (16)	0.0106 (12)	0.0042 (12)	0.0045 (12)
C3	0.0461 (14)	0.0774 (17)	0.0734 (17)	0.0080 (12)	0.0002 (11)	−0.0027 (14)
C4	0.0479 (14)	0.0703 (16)	0.0779 (17)	−0.0168 (12)	0.0061 (12)	−0.0064 (14)
C5	0.0588 (15)	0.0482 (13)	0.0813 (18)	−0.0057 (11)	0.0028 (12)	−0.0127 (12)
C6	0.0449 (12)	0.0551 (13)	0.0632 (14)	0.0039 (10)	0.0108 (10)	−0.0128 (11)
C7	0.0366 (11)	0.0558 (13)	0.0760 (16)	0.0008 (10)	0.0114 (10)	−0.0201 (12)
C8	0.0398 (11)	0.0414 (10)	0.0423 (10)	0.0025 (8)	0.0088 (8)	0.0009 (8)
C9	0.0353 (10)	0.0412 (10)	0.0400 (10)	−0.0013 (8)	0.0078 (8)	−0.0015 (8)
C10	0.0385 (11)	0.0454 (11)	0.0584 (13)	−0.0032 (9)	0.0107 (9)	0.0045 (9)
C11	0.0467 (12)	0.0446 (12)	0.0631 (14)	0.0061 (10)	0.0059 (10)	0.0037 (10)
C12	0.0396 (11)	0.0662 (14)	0.0441 (11)	0.0117 (10)	0.0060 (9)	0.0054 (10)
C13	0.0383 (11)	0.0726 (15)	0.0579 (13)	0.0011 (10)	0.0164 (9)	0.0152 (11)
C14	0.0462 (12)	0.0475 (11)	0.0581 (13)	−0.0004 (10)	0.0140 (10)	0.0111 (10)
C15	0.0564 (16)	0.103 (2)	0.0723 (17)	0.0339 (15)	0.0197 (13)	0.0177 (16)
N1	0.0375 (9)	0.0439 (9)	0.0546 (10)	0.0010 (7)	0.0085 (7)	−0.0041 (8)
N2	0.0355 (9)	0.0418 (9)	0.0729 (12)	0.0023 (7)	0.0118 (8)	−0.0075 (9)
O1	0.0545 (10)	0.0403 (8)	0.0941 (13)	0.0015 (7)	0.0270 (9)	−0.0002 (8)
O1W	0.0542 (10)	0.0416 (9)	0.0731 (13)	0.0003 (7)	0.0194 (8)	−0.0004 (8)

### Geometric parameters (Å, º)

**Table:**

C1—N1	1.461 (3)	C7—H7B	0.9700
C1—C2	1.515 (3)	C8—O1	1.231 (2)
C1—H1A	0.9700	C8—N2	1.328 (3)
C1—H1B	0.9700	C8—C9	1.493 (3)
C2—C3	1.504 (4)	C9—C14	1.383 (3)
C2—H2A	0.9700	C9—C10	1.383 (3)
C2—H2B	0.9700	C10—C11	1.376 (3)
C3—C4	1.501 (4)	C10—H10	0.9300
C3—H3A	0.9700	C11—C12	1.370 (3)
C3—H3B	0.9700	C11—H11	0.9300
C4—C5	1.498 (3)	C12—C13	1.379 (3)
C4—H4A	0.9700	C12—C15	1.503 (3)
C4—H4B	0.9700	C13—C14	1.378 (3)
C5—N1	1.468 (3)	C13—H13	0.9300
C5—H5A	0.9700	C14—H14	0.9300
C5—H5B	0.9700	C15—H15A	0.9600
C6—N1	1.465 (3)	C15—H15B	0.9600
C6—C7	1.496 (3)	C15—H15C	0.9600
C6—H6A	0.9700	N2—H2	0.8600
C6—H6B	0.9700	O1W—H1W	0.849 (17)
C7—N2	1.451 (3)	O1W—H2W	0.845 (18)
C7—H7A	0.9700		
			
N1—C1—C2	111.57 (19)	C6—C7—H7A	109.6
N1—C1—H1A	109.3	N2—C7—H7B	109.6
C2—C1—H1A	109.3	C6—C7—H7B	109.6
N1—C1—H1B	109.3	H7A—C7—H7B	108.2
C2—C1—H1B	109.3	O1—C8—N2	122.92 (18)
H1A—C1—H1B	108.0	O1—C8—C9	120.65 (18)
C3—C2—C1	111.0 (2)	N2—C8—C9	116.43 (17)
C3—C2—H2A	109.4	C14—C9—C10	117.78 (18)
C1—C2—H2A	109.4	C14—C9—C8	119.21 (18)
C3—C2—H2B	109.4	C10—C9—C8	122.98 (17)
C1—C2—H2B	109.4	C11—C10—C9	120.89 (19)
H2A—C2—H2B	108.0	C11—C10—H10	119.6
C4—C3—C2	110.3 (2)	C9—C10—H10	119.6
C4—C3—H3A	109.6	C12—C11—C10	121.6 (2)
C2—C3—H3A	109.6	C12—C11—H11	119.2
C4—C3—H3B	109.6	C10—C11—H11	119.2
C2—C3—H3B	109.6	C11—C12—C13	117.63 (19)
H3A—C3—H3B	108.1	C11—C12—C15	121.2 (2)
C5—C4—C3	111.5 (2)	C13—C12—C15	121.2 (2)
C5—C4—H4A	109.3	C14—C13—C12	121.4 (2)
C3—C4—H4A	109.3	C14—C13—H13	119.3
C5—C4—H4B	109.3	C12—C13—H13	119.3
C3—C4—H4B	109.3	C13—C14—C9	120.7 (2)
H4A—C4—H4B	108.0	C13—C14—H14	119.7
N1—C5—C4	111.6 (2)	C9—C14—H14	119.7
N1—C5—H5A	109.3	C12—C15—H15A	109.5
C4—C5—H5A	109.3	C12—C15—H15B	109.5
N1—C5—H5B	109.3	H15A—C15—H15B	109.5
C4—C5—H5B	109.3	C12—C15—H15C	109.5
H5A—C5—H5B	108.0	H15A—C15—H15C	109.5
N1—C6—C7	112.87 (18)	H15B—C15—H15C	109.5
N1—C6—H6A	109.0	C1—N1—C6	112.36 (18)
C7—C6—H6A	109.0	C1—N1—C5	109.23 (18)
N1—C6—H6B	109.0	C6—N1—C5	108.86 (17)
C7—C6—H6B	109.0	C8—N2—C7	123.92 (18)
H6A—C6—H6B	107.8	C8—N2—H2	118.0
N2—C7—C6	110.08 (18)	C7—N2—H2	118.0
N2—C7—H7A	109.6	H1W—O1W—H2W	103 (2)
			
N1—C1—C2—C3	−56.8 (3)	C11—C12—C13—C14	0.0 (3)
C1—C2—C3—C4	52.5 (3)	C15—C12—C13—C14	−179.8 (2)
C2—C3—C4—C5	−53.0 (3)	C12—C13—C14—C9	−1.3 (4)
C3—C4—C5—N1	57.1 (3)	C10—C9—C14—C13	1.8 (3)
N1—C6—C7—N2	177.01 (19)	C8—C9—C14—C13	179.7 (2)
O1—C8—C9—C14	−19.8 (3)	C2—C1—N1—C6	−179.81 (19)
N2—C8—C9—C14	159.4 (2)	C2—C1—N1—C5	59.3 (3)
O1—C8—C9—C10	157.9 (2)	C7—C6—N1—C1	77.8 (3)
N2—C8—C9—C10	−22.9 (3)	C7—C6—N1—C5	−161.1 (2)
C14—C9—C10—C11	−1.1 (3)	C4—C5—N1—C1	−59.5 (3)
C8—C9—C10—C11	−178.91 (19)	C4—C5—N1—C6	177.5 (2)
C9—C10—C11—C12	−0.1 (3)	O1—C8—N2—C7	−1.7 (3)
C10—C11—C12—C13	0.7 (3)	C9—C8—N2—C7	179.16 (19)
C10—C11—C12—C15	−179.4 (2)	C6—C7—N2—C8	133.2 (2)

### Hydrogen-bond geometry (Å, º)

**Table:**

*D*—H···*A*	*D*—H	H···*A*	*D*···*A*	*D*—H···*A*
O1*W*—H1*W*···O1^i^	0.85 (2)	1.99 (2)	2.840 (2)	177 (3)
O1*W*—H2*W*···N1^ii^	0.85 (2)	2.04 (2)	2.883 (3)	177 (3)
N2—H2···O1*W*	0.86	2.11	2.906 (2)	153
C7—H7*A*···O1*W*^iii^	0.97	2.55	3.472 (3)	159
C7—H7*A*···O1	0.97	2.44	2.812 (3)	102
C10—H10···O1*W*	0.93	2.51	3.374 (3)	154

Symmetry codes: (i) *x*, *y*−1, *z*; (ii) −*x*+1, *y*−1/2, −*z*+1/2; (iii) *x*, *y*+1, *z*.
