# Supplementary figures and images for: Crystal structure of 4-methyl-N-[2-(piperidin-1-yl)eth­yl]benzamide monohydrate
Source: Acta Crystallogr E Crystallogr Commun. 2015 Apr 30;71(Pt 5):o359–60. doi: 10.1107/S2056989015007653 (PMC4420061; doi:10.1107/S2056989015007653)

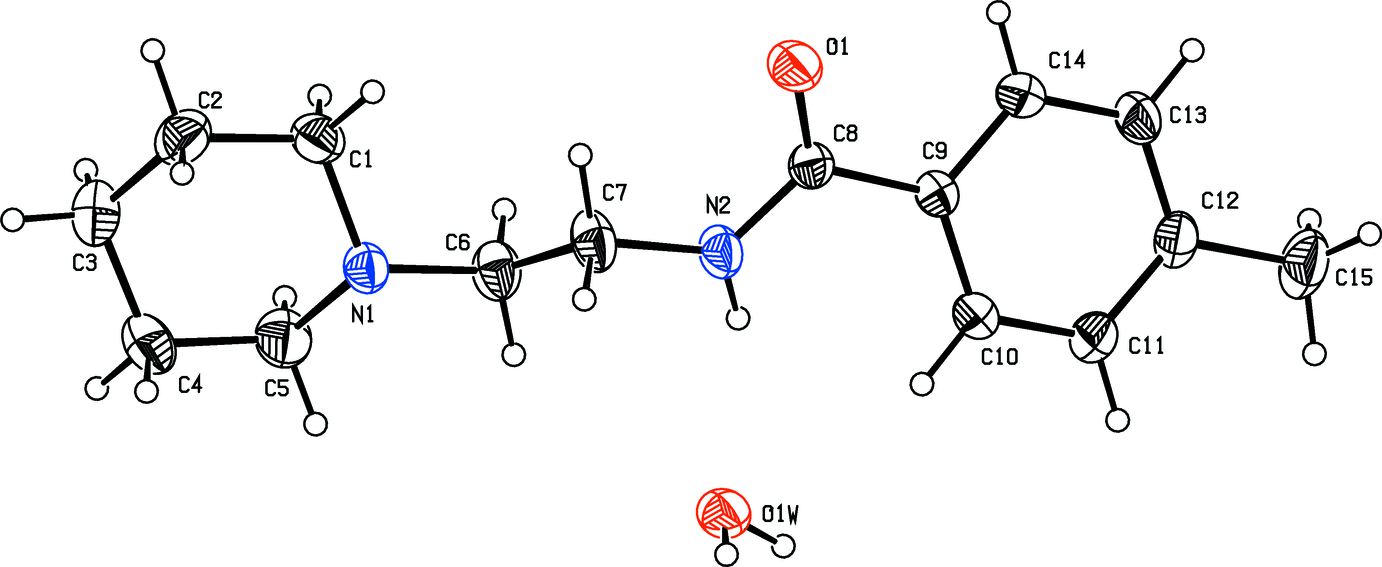

Supplement: Supplementary file 4 [file e-71-0o359-fig1.tif]

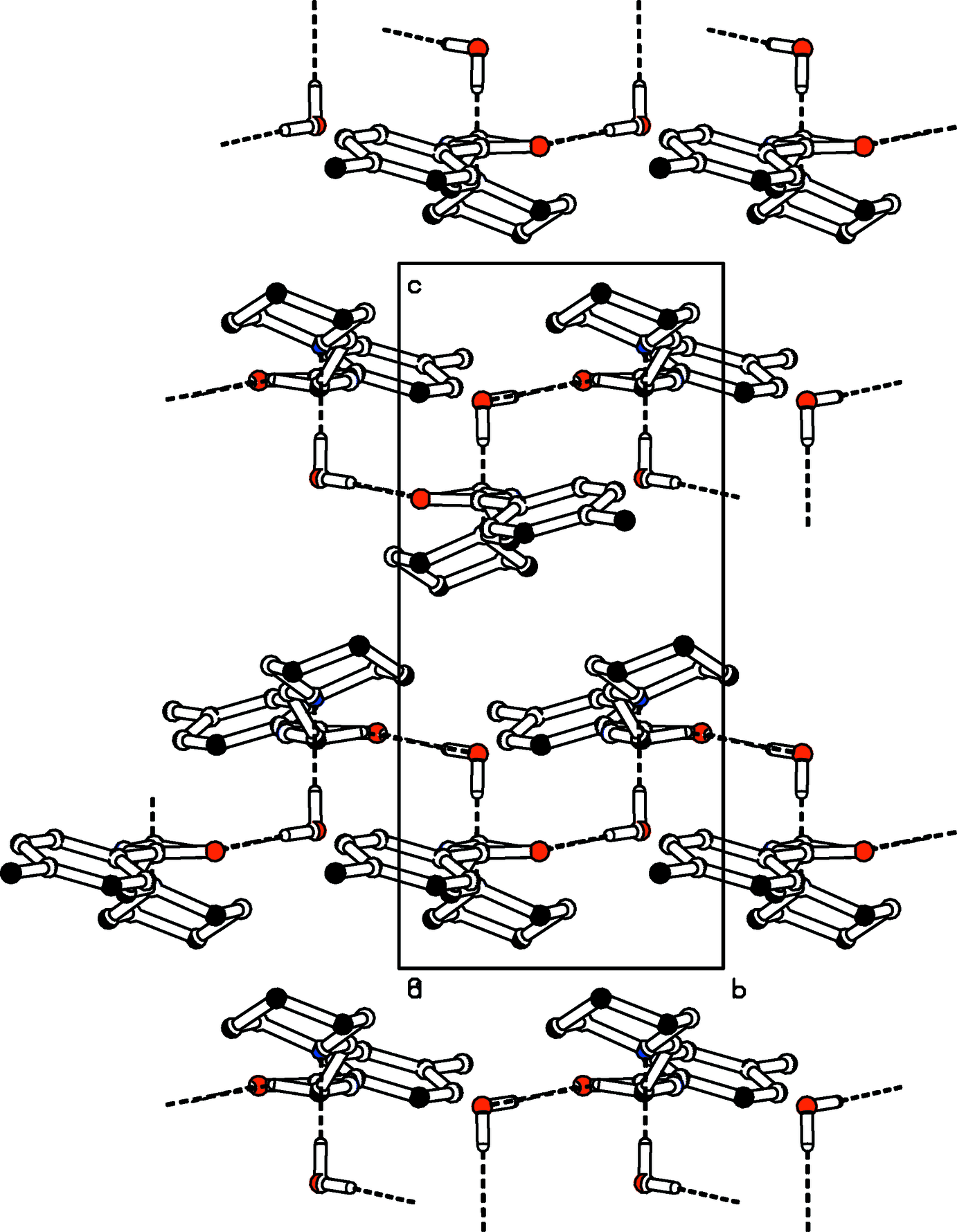

Supplement: Supplementary file 5 [file e-71-0o359-fig2.tif]
